# Vitamin D Regulates Maternal T-Helper Cytokine Production in Infertile Women

**DOI:** 10.3390/nu10070902

**Published:** 2018-07-13

**Authors:** Yuko Ikemoto, Keiji Kuroda, Koji Nakagawa, Asako Ochiai, Rie Ozaki, Keisuke Murakami, Makoto Jinushi, Akemi Matsumoto, Rikikazu Sugiyama, Satoru Takeda

**Affiliations:** 1 Department of Obstetrics and Gynecology, Juntendo University, Faculty of Medicine, Tokyo 1138421, Japan; camomile1013@hotmail.co.jp (Y.I.); anishino@juntendo.ac.jp (A.O.); rieozaki@juntendo.ac.jp (R.O.); kmuraka@juntendo.ac.jp (K.M.); makoto-j@juntendo.ac.jp (M.J.); amatsu@juntendo.ac.jp(A.M.); stakeda@juntendo.ac.jp (S.T.); 2 Center for Reproductive Medicine and Implantation Research, Sugiyama Clinic Shinjuku, Tokyo 1160023, Japan; nakagawa-jiko@spice.ocn.ne.jp (K.N.); riki@sugiyama.or.jp (R.S.)

**Keywords:** vitamin D, preconception care, helper T cell, infertility

## Abstract

Vitamin D (VD) deficiency is associated with reproductive failure. However, the relationship between VD and maternal immunity remains unclear. We investigated the clinical efficacy of VD in maternal T-helper (Th) cytokines in 276 infertile women and examined for Th1 and Th2 cells based on the deficient, insufficient, and sufficient serum 25-hydroxyvitamin D3 (25[OH]VD) levels (<12, 12–30, and >30 ng/mL, respectively). Most infertile women had a low-level of VD (87.3%). Immunological tests of pre-/post-VD supplementation were performed in patients who were deficient and insufficient in VD. Of 23 patients, 11 (47.8%) exhibited sufficient VD levels after supplementation. Th1/Th2 cell ratio in patients with insufficient VD was significantly decreased after supplementation (*p* = 0.004). After supplementation, serum 25(OH)VD levels of the patients: 11 in the sufficient group showed significant decreases in Th1 cell level and Th1/Th2 cell ratio (*p* = 0.032 and 0.010, respectively), whereas no significant differences in Th1/Th2 cell ratio were recognized in the insufficient group. Furthermore, mid-luteal endometrial biopsies (*n* = 18) were processed for primary cultures and measured interferon [IFN]-γ and interleukin [IL]-4 in condition media. Decidualizing cultures with 1,25-dihydroxvitamin D3 (1,25[OH]_2_VD) decreased IFN-γ. Sufficient VD supplementation in women with insufficient VD may optimize maternal T-helper cytokines during pregnancy via rebalancing the Th1/Th2 cell ratio.

## 1. Introduction

Vitamin D (VD) is widely known as a steroid hormone associated with bone metabolism and calcium homeostasis [1]. VD also modulates immune function, such as T-helper (Th) cells, in a variety of organ systems [2]. As such, VD deficiency links with many autoimmune diseases that include systemic lupus erythematosus, type 1 diabetes, and autoimmune thyroid disease [3,4,5]. Insufficient serum VD is also associated with reproductive failure, which includes the implantation failure after in vitro fertilization (IVF) and embryo transfer (ET), and pregnancy complications, such as a recurrent miscarriage, preeclampsia, and gestational diabetes mellitus [6,7,8,9]. Rudick B. et al. [9] reported that recipient infertile patients with VD deficiency in an oocyte donation program had low implantation rates after IVF-ET.

Successful pregnancy requires an attachment of a semiheterograft embryo in the decidualized endometrium. Maternal immune tolerance in pregnancy is associated with balancing of Th cells, including Th1 (interleukin [IL]-2, intracellular interferon [IFN]-γ production), and Th2 (IL-4, IL-5, and IL-10 production) cells, in favor of Th2 cells [10,11]. Thus, impaired Th1- and Th2- cell balance can cause the reproductive failure, such as infertility and miscarriage.

The regulatory T (Treg) cell is known as an immune inhibitor of perturbed activation of immune responses against self-antigens. In human decidua, Treg cells are increased during the peri-implantation window locally because of a regulation of immune cytotoxicity against the conceptus antigen [12]. Human spontaneous abortion is involved with a decrease in Treg cells [13]. Besides, Th17 cells induce the chronic inflammatory processes with the production of IL-17 [14]. In human peripheral blood and decidua, the aberrant Th17 cell-mediated inflammatory response is related to a recurrent miscarriage [15]. As such, the optimal balance of Th1 and Th2 cells, as well as Treg and Th17 cells are important for successful pregnancy [10,11,16].

The storage form of VD; i.e., 25-hydroxyvitamin D_3_ (25[OH]VD), is converted to a requisite small amount of 1,25-dihydroxvitamin D_3_ (1,25[OH]_2_VD) by 25-hydroxyvitamin d-1α-hydroxylase (CYP27B1) at the target organ in vivo. The main cellular target for 1,25(OH)_2_VD is the vitamin D receptor (VDR), which is present in most tissues and cells in the body [17,18,19,20,21]. VDR-null mutant mice showed significant gonadal insufficiencies that led to hypergonadotropic hypogonadism with a reduction of activity of aromatase in the ovary [22]. Therefore, VD is an essential factor for a full gonadal function. In an in vitro study, VD treatment regulates the T-helper cell populations through an inhibition of cytotoxic Th1 cell proliferation, promotion of Th2 cells, suppression of Th17, and the induction of Treg cells in other tissues [23,24,25]. In the human uterus, local decidualized cells synthesize active 1,25(OH)_2_VD during early pregnancy [21]. Human decidua treated with 1,25(OH)_2_VD decreased and increased the expressions of Th1 cytokine (*IFNG*) and antimicrobial peptide (*CAMP*), respectively [21], suggesting that VD was an important suppressor of cytotoxic immune cytokines. However, the immunomodulatory effect of VD on pregnancy and the optimal preconception VD level remain poorly understood.

We evaluated the relationship between VD status and T-helper cells in infertile women and identified the role of VD in embryo receptivity via investigation of maternal T-helper cytokines.

## 2. Materials and Methods

### 2.1. Patient Selection

This study was approved by the local ethics committee of Juntendo University Faculty of Medicine (No. 14-103, Tokyo, Japan) and Sugiyama Clinic (No. 16-002; Tokyo, Japan). Blood and endometrial cell samples were collected after an informed written consent. Of 589 consecutive infertile women who underwent immunologic examinations as a test for the repeated reproductive failure between September 2014 and December 2017, we excluded 313 with potential impact factors on their immunity, including the use of immunosuppressive drugs (*n* = 4) and potential reproductive failure with immune abnormality, and recurrent miscarriage (*n* = 68) with a history of more than or equal to three times of clinical miscarriages and repeated implantation failure (*n* = 248), defined by more than or equal to four times of implantation failures after ET with morphologically good-quality embryos. Seven women had a history of recurrent miscarriage as well as repeated implantation failure. The storage form of VD, i.e., 25(OH)VD level is the best parameter to evaluate the VD status. In the final recruited 276 general infertile patients, we compared peripheral blood Th1 and Th2 cell levels and Th1/Th2 cell ratio among those with deficient, insufficient, and sufficient serum 25(OH)VD levels (<12, 12–30, and ≥30 ng/mL, respectively), according to a previous report [26].

### 2.2. Vitamin D Prospective Intervention Study

We analyzed alteration of T-helper cells in patients with VD deficiency before and after supplementation for 3 months. Of 28 infertile patients (age ≤40 years) with ≤30 ng/mL 25(OH)VD enrolled from the Fertility Outpatient Clinic, Department of Obstetrics and Gynecology, Juntendo University Hospital, five were excluded because one was absent during the follow-up, and four used immunosuppressive drugs because of post-liver transplantation or collagen disease. The remaining 23 patients received Vitamin D 1000 (Douglas Laboratories Company, Pittsburgh, PA, USA), which contained vitamin D_3_ (cholecalciferol), at a dose of 1000 IU per day for 3 months. Changes in serum VD status and various T-helper cell levels were analyzed before and after supplementation.

### 2.3. Analysis of T-helper Cells

Venous blood samples were obtained from the infertile patients for an evaluation of T-helper cells. Th1, Th2, Th17, and Treg cells were defined by measuring the IFN-γ, IL-4, IL-17, and the forkhead box P3 protein (FoxP3) production, respectively. We consigned flow cytometry to the company, SRL Inc., Tokyo, Japan. Blood samples were analyzed on the sampling day by laser flow cytometry (Fascinator II; BD Biosciences, Franklin Lakes, NJ, USA) using Phorbol 12-Myristate 13 Acetate, Ionomycin, Brefeldin-A (Sigma-Aldrich Corp., St. Louis, MO, USA), CD4 R-phycoerythrin-cyanine [PC]-5 (Immunotech, Oxford, UK), Fluorescence activated cell sorting [FACS] Lysing Solution (BD Biosciences), FACS Permeabilizing Solution 2 (BD Biosciences), Fastimmune IFN-γ, and Fluorescein isothiocyanate [FITC]/IL-4 PE (BD Biosciences). After surface staining of the activated whole blood samples with anti-CD4–PC5-conjugated monoclonal antibodies, red blood cell lysis and specific intracellular staining using FastImmuneTM IFN-γ-FITC/IL-4-PE (Becton Dickinson Biosciences, San Jose, CA, USA) were subsequently performed according to the manufacturer’s instructions. Th1 cells were determined as CD4^+^ T lymphocytes with IFN-γ without IL-4. Moreover, Th2 cells were CD4^+^ T lymphocytes with IL-4 without IFN-γ. The ratio of Th1/Th2 cell ratio was IFN-γ- to IL-4–positive T-helper cells. Th17 cells were CD4^+^ T lymphocytes with IL-17. In addition, Treg cells were CD4^+^, CD25^+^, and FoxP3^+^ T cells. Flow cytometry analysis was performed with FlowJo software (FlowJo ver.10; LLC, Ashland, OR, USA).

### 2.4. Analysis of Vitamin D

Serum 25(OH)VD and 1,25(OH)_2_VD levels were analyzed with double-antibody radioimmunoassay (SRL Inc, Tokyo, Japan) using the cryopreservation blood serum samples. 25(OH)VD concentration was measured by γ-counter (ARC-950; Hitachi-Aloka Medical, Tokyo, Japan) using the 25-Hydroxyvitamin D 125 I RIA Kit (Sceti Medical Labo K.K., Tokyo, Japan) and Acetonitrile 300 (Wako Pure Chemical Industries Ltd., Osaka, Japan). Moreover, 1,25(OH)_2_VD was measured by γ-counter (ARC-950, Hitachi-Aloka Medical) using the 1,25-Dihydroxy Vitamin D RIA (Immunodiagnostic Systems, East Boldon, UK).

### 2.5. Cytokine Analysis in Primary Human Endometrial Cell Culture

Endometrial samples were collected 7–11 days after luteinizing hormone surge from premenopausal infertile volunteers (*n* = 18) without endometriosis, intrauterine disorder, and any hormonal therapy. Human endometrial stromal cells (HESCs) were isolated, cultured, and maintained as described previously [27]. Primary cultures were passed once, allowed to grow to confluency and decidualized with 0.5 mM 8-bromoadenosine 3′5′-cyclic adenosine monophosphate (8-bromo-cAMP; B7880; SIGMA, Kanagawa, Japan), 1 μM progesterone (P4; P0130, SIGMA) with or without 1.2 × 10^−7^ M 1,25(OH)_2_VD (#C0145; LKT Labs, St. Paul, MN, USA) for 4 days. In accordance with the previous studies, the concentration of 1.0–1.2 × 10^−7^ M for 1,25(OH)_2_VD was selected as the appropriate physiologic level of circulating VD [28,29]. Secreted cytokine IFN-γ and IL-4 levels in culture media of HESCs with 8-bromo-cAMP and P4 with or without 1.2 × 10^−7^ M 1,25(OH)_2_VD were measured using the specific capture enzyme-linked immunosorbent assay (ELISA) sets (IFN-γ 550612, IL-4 550614; Human OptEIATMELISA Kit; BD, San Diego, CA, USA) according to the manufacturer’s instructions.

### 2.6. Immunohistochemical Staining for Vitamin D Receptor

HESCs were seeded in four-well chamber slides and cultured until confluency with 8-bromo-cAMP and P4 with or without 1.2 × 10^−7^ M 1,25(OH)_2_VD and then fixed with 4% paraformaldehyde. As such, the endogenous peroxide activity was inactivated with 1% H_2_O_2_ and blocked by 2% bovine serum albumin/Tris-buffered saline. After removing blocking buffer, the HESCs were stained with primary anti-VD receptor antibody (1:500, ab8756; Abcam, Inc., Cambridge, UK) at 4 °C overnight. After rinsing the cells, biotinylated rabbit anti-rat IgG (1:300, BA-4001; Vector Laboratories, Burlingame, CA, USA) was added and the cells were incubated at room temperature. VDR expression was detected using horseradish peroxidase-conjugated streptavidin (1:300, P0397; DAKO Japan, Tokyo, Japan). Staining was developed with 3,3′-diaminobenzidine (WAKO, Osaka, Japan), followed by counterstaining with hematoxylin and washing with cold running water. The primary antibody was omitted in the negative controls. Digital images were acquired using a section microscope scanner (BZ-X700, Keyence, Osaka, Japan).

### 2.7. Statistical Analysis

Categorical variables were evaluated with the Kruskal-Wallis test or chi-square test as appropriate. Spearman’s rank correlation coefficient was used to evaluate the correlation of 25(OH)VD and helper T cells. Student’s *t*-test was used to compare the differences between pre- and post-VD supplementation data. All statistical analyses were performed with GraphPad Prism ver.6.07 for Windows (GraphPad Software, San Diego, CA, USA). Statistical significance was defined as *p* < 0.05.

## 3. Results

### 3.1. Status of Helper T-Cell Immunity and Vitamin D in Infertile Women

The clinical characteristics of the infertile patients are shown in the Supplementary Table S1. Age and serum anti-Müllerian hormone level of the total study population were 36.0 ± 3.6 years and 3.7 ± 3.5 ng/mL, respectively. Of the patients, 18 (6.5%), 223 (80.8%), and 35 (12.7%) had VD deficiency, insufficiency, and sufficiency, respectively (Table 1). Most infertile women (87.3%) had 25(OH)VD level below 30 ng/mL. Immunologic profiles of the infertile women with VD deficiency, insufficiency, and sufficiency were shown in Table 1.

**Figure 1:**
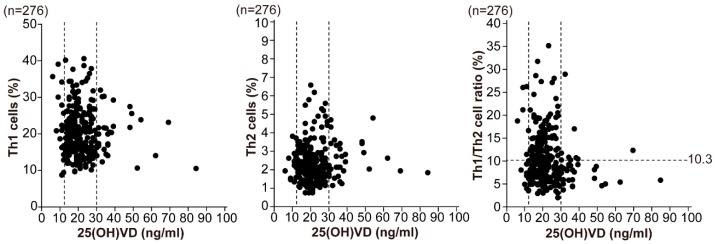
Relationship between serum 25(OH)VD and T-helper (Th) cells in infertile patients. The data demonstrated the relationships between 25(OH)VD and the Th1 and Th2 cell populations, and the Th1/Th2 cell ratio. All data were divided into three groups that were categorized as vitamin D deficiency, insufficiency, and sufficiency (25[OH]VD < 12, 12–30, and > 30 ng/mL respectively; *dotted lines*). Regarding the Th1 (CD4^+^/interferon [IFN]-γ^+^)/Th2 (CD4^+^/interleukin[IL]-4^+^) cell ratio, the normal range is <10.3 (*dotted lines*). Analysis of data from 276 general infertile patients showed a higher Th1/Th2 cell ratio in the VD-deficient and insufficient groups, but no correlation between serum 25(OH)VD and Th1 or Th2 cells or Th1/Th2 cell ratio. 25(OH)VD, 25-hydroxy vitamin D3; Th1 cell, IFN-γ–producing T-helper cell (CD4^+^/IFN-γ^+^); Th2 cell, IL-4–producing T-helper cell (CD4^+^/IL-4^+^).

Th1/Th2 cell ratio in the sufficient group was relatively lower, but there was no significant difference among the three groups. There was no correlation between serum 25(OH)VD level and Th1 or Th2 cells, or Th1/Th2 cell ratio among the 276 general infertile patients (Figure 1). Nakagawa K. et al. [30] demonstrated that the aberrant elevated Th1 (CD4^+^/IFN-γ^+^)/Th2 (CD4^+^/IL-4^+^) cell ratio in women with a history of normal delivery was more than 10.3. Although there were no significant differences in Th1 cell level and Th1/Th2 cell ratio among sufficient, insufficient and deficient groups, our results showed that 41.9% (101/241 women) of patients with the VD insufficient and deficient groups had 10.3 or more in Th1/Th2 cell ratios, with a significantly higher rate comparing to that in the sufficient group (20.0%, 7/35 women, *p* = 0.046).

### 3.2. Immunological Impact of Preconception Vitamin D Supplementation

**Figure 2:**
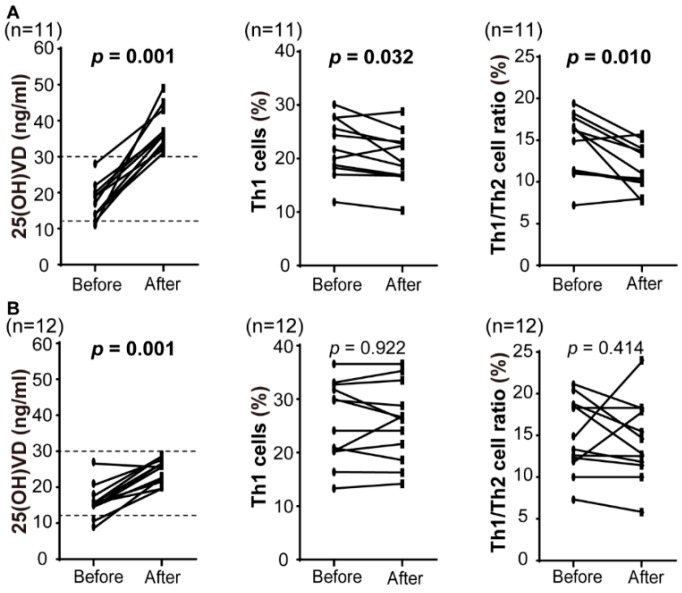
Immunological alteration after VD supplementation in infertile women. A change in serum 25(OH)VD, Th1 cell, and Th1/Th2 cell ratio after intake of VD (cholecalciferol) at 1000 IU per day for 3 months in the infertile patients with VD insufficiency or deficiency. Normal range of Th1 (CD4^+^/IFN-γ^+^)/Th2 (CD4^+^/IL-4^+^) cell ratio is <10.3 (*dotted lines*). Of 23 patients, 11 reached above normal range after supplementation, whereas Th1 and Th1/Th2 ratio were significantly reduced (**A**). However, 12 patients who did not reach normal range showed no significant difference (**B**). 25(OH)VD, 25-hydroxyvitamin D_3_; Th1 cell, IFN-γ–producing T-helper cell (CD4^+^/IFN-γ^+^); Th2 cell, IL-4–producing T-helper cell (CD4^+^/IL-4^+^).

To identify the effect of the VD intervention on the systemic immune responses, we examined serum VD and T-helper cell levels before and after VD supplementation in patients with VD insufficiency or deficiency (Table 2). Intake of VD (cholecalciferol) at 1000 IU daily for 3 months significantly increased to 25(OH)VD levels (16.7 ± 4.7 to 31.0 ± 7.8 ng/mL, *p* < 0.0001). Of 23 patients, 22 (95.7%) recorded an increase of 25(OH)VD, and 11 (47.8%) reached sufficient VD levels (>30 ng/mL). VD supplementation relatively decreased and increased Th1 and Th2 cell levels, respectively, resulting in a significant inhibition of the Th1/Th2 cell ratio (14.8 ± 4.0 to 13.1 ± 4.1, *p* = 0.004). The biologically active form, 1,25(OH)_2_VD level was significantly increased (*p* = 0.019). Th17 and Treg cell levels were not changed significantly after VD supplementation (*p* = 0. 315 and *p* = 0.059, respectively; Table 2). We divided the patients into two groups with sufficient and insufficient groups after VD supplementation (Figure 2). Eleven patients in the sufficient group showed a significant decrease in Th1 cell level and Th1/Th2 cell ratio (*p* = 0.032 and 0.010, respectively), whereas no significant differences in Th1/Th2 cell ratio were recognized in the insufficient group. No complications or adverse effects of VD supplementation were identified in any patient.

### 3.3. Effect of Vitamin D on Decidualized Endometrial Stromal Cells

**Figure 3:**
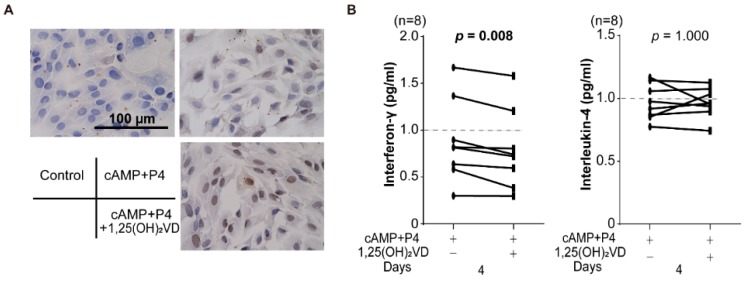
Role of vitamin D in decidualization of human endometrial stromal cell. (**A**) Immunohistochemical staining for vitamin D receptor (VDR) in human endometrial stromal cells [HESCs]. VDR was accumulated in the nucleus of HESCs cultured with 8-bromoadenosine 3′5′-cyclic adenosine monophosphate [8-bromo-cAMP] + P4 + 1,25(OH)_2_VD. (**B**) Cytokine assay of culture media of HESCs with 8-bromo-cAMP and P4 with or without 1,25(OH)_2_VD. IFN-γ levels were significantly reduced in decidualized cells with compared to those without 1,25(OH)_2_VD. * *p* < 0.05.

To identify the location of VDR, immunohistochemical staining for VDR in undifferentiated HESCs, and cells decidualized with 8-bromo-cAMP and P4 with or without 1,25(OH)_2_VD treatment for 4 days demonstrated that decidualization is associated with diffuse distribution of VDR in the cytoplasm in the cells. In decidualized cells with 1,25(OH)_2_VD treatment, VDR accumulated in the nucleus compared to the cells without 1,25(OH)_2_VD treatment (Figure 3A). Cytokine assay of condition media of decidualized HESCs with or without 1,25(OH)_2_VD demonstrated that IFN-γ, but not IL-4, levels were significantly reduced in decidualized cells compared to those without 1,25(OH)_2_VD treatment (*p* = 0.008; Figure 3B). We confirmed decidualization of HESCs using decidual markers, insulin-like growth factor-binding protein-1 [*IGFBP1*] and prolactin [*PRL*] mRNA expression (Supplementary Figure S1).

## 4. Discussion

Epidemiologically, the rate of reproductive-aged women with VD insufficiency and deficiency has been reported to be 20–90%, with a significantly higher rate between 2000 and 2004 compared with the 1990s rate [31]. Our study demonstrated that most infertile women had insufficient 25(OH)VD levels (Table 1). Indeed, average serum 25(OH)VD levels in subfertile women were reportedly lower than those in normal fertile women [32,33]. In northern European countries, seasonal variation in conception rates was attributed to changes in the VD levels with the sunlight exposure; as such, VD status was strongly associated with the conception rate [34]. VD deficiency is a global health issue in reproductive age women [35,36].

An aberrantly high Th1/Th2 cell ratio during the preconception period leads to the reproductive failure [30,37]. Our results showed that 41.9% of Japanese infertile patients with the VD insufficient and deficient groups had 10.3 or more in Th1/Th2 cell ratios, suggesting that nearly half of the infertile patients without sufficient VD levels had impaired immunologic tolerance. According to other reports, VD regulates T-helper cell populations through an inhibition of Th1 cell proliferation and promotion of Th2 cells [24]. In our data, VD supplementation decreased serum Th1/Th2 cell ratio; however, there was no significant change in each Th1 and Th2 cell level in infertile women with VD insufficiency or deficiency (Table 2). Nevertheless, when focusing on 11 patients who reached sufficient 25(OH)VD levels after supplementation, a decrease in serum Th1 cell level and Th1/Th2 cell ratio was recognized. Therefore, an optimal 25(OH)VD level for supporting pregnancy may be ≥30 ng/mL and may have a significant role in the regulation of immunologic embryo receptivity. In fact, VD replacement for patients with a history of recurrent pregnancy loss improves immune abnormality with insufficient or sufficient VD status, resulting in a prevention of pregnancy loss [38].

At local sites, 1,25(OH)_2_VD treatment promoted expression of VDR in the nucleus. Decidual change in HESCs did not alter secretion of IFN-γ and IL-4 significantly in the culture media. However, treatment of decidualized cells with 1,25(OH)_2_VD decreased the IFN-γ level, like the serum Th1 cell level. VD may have an important role in the regulation of not only systemic but also local Th1 immune response for optimization of maternal tolerance for pregnancy. Although VD has been reported to inhibit the response of Th17 cell levels and induce Treg cells [12,13,25], our results showed that Treg cells were relatively increased by VD supplementation. However, there was no significant difference in Th17 and Treg cell levels (Table 1).

In our study, 1000 IU VD replacement per day for 3 months resulted in sufficient VD levels (>30 ng/mL) in only half of the patients with VD deficiency/insufficiency. A dose of 1000 IU VD replacement per day may be insufficient as preconception care for normalization of maternal VD and immune status. Therefore, a proper dose of VD supplementation for the infertile women without VD sufficiency may be more than 1000 IU. And the infertile women with an elevated Th1/Th2 cell ratio after VD supplementation may need to receive immunotherapy including tacrolimus during IVF-ET treatment [30]. Taken together, proper VD replacement for infertile VD-insufficient patients may contribute to the maintenance of immune homeostasis by suppressing Th1 cells for a successful pregnancy.

This study has some limitations. First, VD is produced in the skin via sunlight exposure. Therefore, serum VD level varied according to the seasonal changes. In our data, no significant difference in the VD levels was recognized among the three groups (summer, winter, and both spring and autumn; Supplementary Table S2). Second, as regarding the experiments of the effect of VD intervention on T-helper cell levels, the number of the patients was 23, thus the study size was small to evaluate the effect of VD supplementation correctly.

## 5. Conclusions

Sufficient VD supplementation in women with insufficient or deficient VD may optimize maternal immune tolerance for pregnancy, via rebalancing the Th1/Th2 cell ratio, leading to prevention against proinflammatory obstetrical complications during pregnancy.

## Figures and Tables

**Table 1:** Relationship between 25(OH)VD and T-helper (Th) cell populations in infertile women.

	Deficiency (*n* = 18)	Insufficiency (*n* = 223)	Sufficiency (*n* = 35)	*p* Value
Age (years)	35.6 ± 4.6	36.1 ± 3.6	35.7 ± 3.5	0.899
25(OH)VD (ng/mL)	10.7 ± 1.7	20.2 ± 4.4	39.3 ± 12.3	**<0.0001**
Th1 cells (%)	21.6 ± 8.9	20.8 ± 6.5	19.6 ± 5.6	0.748
Th2 cells (%)	2.3 ± 0.7	2.3 ± 1.0	2.5 ± 1.0	0.621
Th1/Th2 cell ratio	10.9 ± 7.5	10.6 ± 5.6	8.6 ± 4.5	0.411

Values are average ± standard deviation; 25(OH)VD, 25-hydroxyvitamin D_3_ (deficiency: <12 ng/mL, insufficiency: 12–30 ng/mL, sufficiency: >30 ng/mL); Th1 cell, interferon [IFN-γ]-producing T-helper cell (CD4^+^/IFN-γ^+^); Th2 cell, interleukin(IL)-4-producing T-helper cell (CD4^+^/IL-4^+^).

**Table 2:** Impact of vitamin D supplementation for infertile patients with vitamin D insufficiency and deficiency.

*n* = 23	Before	After	*p* Value
25(OH)VD (ng/mL)	16.7 ± 4.7	31.0 ± 7.8	**<0.0001**
1,25(OH)_2_VD (pg/mL)	49.9 ± 8.2	65.4 ± 16.8	**0.019**
Th1 cells (%)	24.0 ± 6.7	23.0 ± 6.8	0.135
Th2 cells (%)	1.7 ± 0.6	1.9 ± 0.6	0.141
Th1/Th2 cell ratio	14.8 ± 4.0	13.1 ± 4.1	**0.004**
Th17 cells (%)	1.65 ± 0.40	1.54 ± 0.63	0.315
Treg cells (%)	5.74 ± 0.98	6.38 ± 1.03	0.059

Values are average ± standard deviation. 25(OH)VD, 25 hydroxyvitamin D_3_; 1,25(OH)_2_VDl 1,25 dihydroxyvitamin D_3_; Th1 cell, interferon [IFN]-γ-producing T-helper cell (CD4^+^/IFN-γ^+^/interleukin[IL]-4^−^); Th2 cell, IL-4-producing T-helper cell (CD4^+^/IFN-γ^-^/IL-4^+^); Th17 cell, IL-17-producing T-helper cell (CD4^+^/IL-17^+^); Reguratory T [Treg] cell, CD4^+^/CD25^+^/FoxP3 T-helper cell. 1,25(OH)2VD, Th17 cells, and Treg cells were measured in eight patients.

## Supplementary Materials

The following are available online at http://www.mdpi.com/2072-6643/10/7/902/s1, Figure S1: RTQ-PCR analysis of *IGFBP1* and *PRL* transcript levels in HESCs treated with 8-bromo-cAMP and P4 combined with or without 1,25(OH)_2_VD for 4 days, Table S1: Patient characteristics, Table S2: Relationship of 25(OH)VD and season.
