# Supplementary material for: Vitamin D Regulates Maternal T-Helper Cytokine Production in Infertile Women
Source: Nutrients. 2018 Jul 13;10(7):902. doi: 10.3390/nu10070902 (PMC6073370; doi:10.3390/nu10070902)
Supplement: Supplementary file 1 [file nutrients-10-00902-s001.zip › Supplemental tableS1.docx]

**Supplemental table S1.** Patient characteristics

| No. of patients | 276 |
| --- | --- |
| Age (y) | 36.0 ± 3.6 |
| History of pregnancy (*n*)  Gravidity  Parity | 0 (0―4)  0 (0―3) |
| AMH (ng/mL) | 3.7 ± 3.5 |
| Causes of infertility *n* (%)  Tubal factor  Endometriosis  Ovulation disorder  Uterus factor  Male factor  Ovarian factor  Unexplained infertility  (2 factors | 30 (10.9)  21 (7.6)  9 (3.3)  2 (0.7)  112 (40.6)  17 (6.2)  115 (41.7)  30) |

Values are average ± standard deviation or median (range) or number (%).

AMH, anti-Müllerian hormone.
