# Supplementary material for: Vitamin D Regulates Maternal T-Helper Cytokine Production in Infertile Women
Source: Nutrients. 2018 Jul 13;10(7):902. doi: 10.3390/nu10070902 (PMC6073370; doi:10.3390/nu10070902)
Supplement: Supplementary file 1 [file nutrients-10-00902-s001.zip › Supplemental tableS2.docx]

**Supplemental table S2.** Relationship of 25(OH)VD and season

|  | Summer  (Jun.―Aug. *n* = 69) | Winter  (Dec.―Feb. *n* = 67) | Spring and Autumn  (Mar.―May and  Sep.–Nov. *n* = 140) | *P* value |
| --- | --- | --- | --- | --- |
| Age (y) | 36.0 ± 3.7 | 35.6 ± 3.5 | 36.2 ± 3.7 | 0.438 |
| 25(OH)VD (ng/mL) | 21.8 ± 9.7 | 22.2 ± 10.6 | 21.9 ± 8.1 | 0.938 |

Values are average ± standard deviation.

25(OH)VD: 25-hydroxyvitamin D_3_.
