# Supplementary figures and images for: Vitamin D Regulates Maternal T-Helper Cytokine Production in Infertile Women
Source: Nutrients. 2018 Jul 13;10(7):902. doi: 10.3390/nu10070902 (PMC6073370; doi:10.3390/nu10070902)

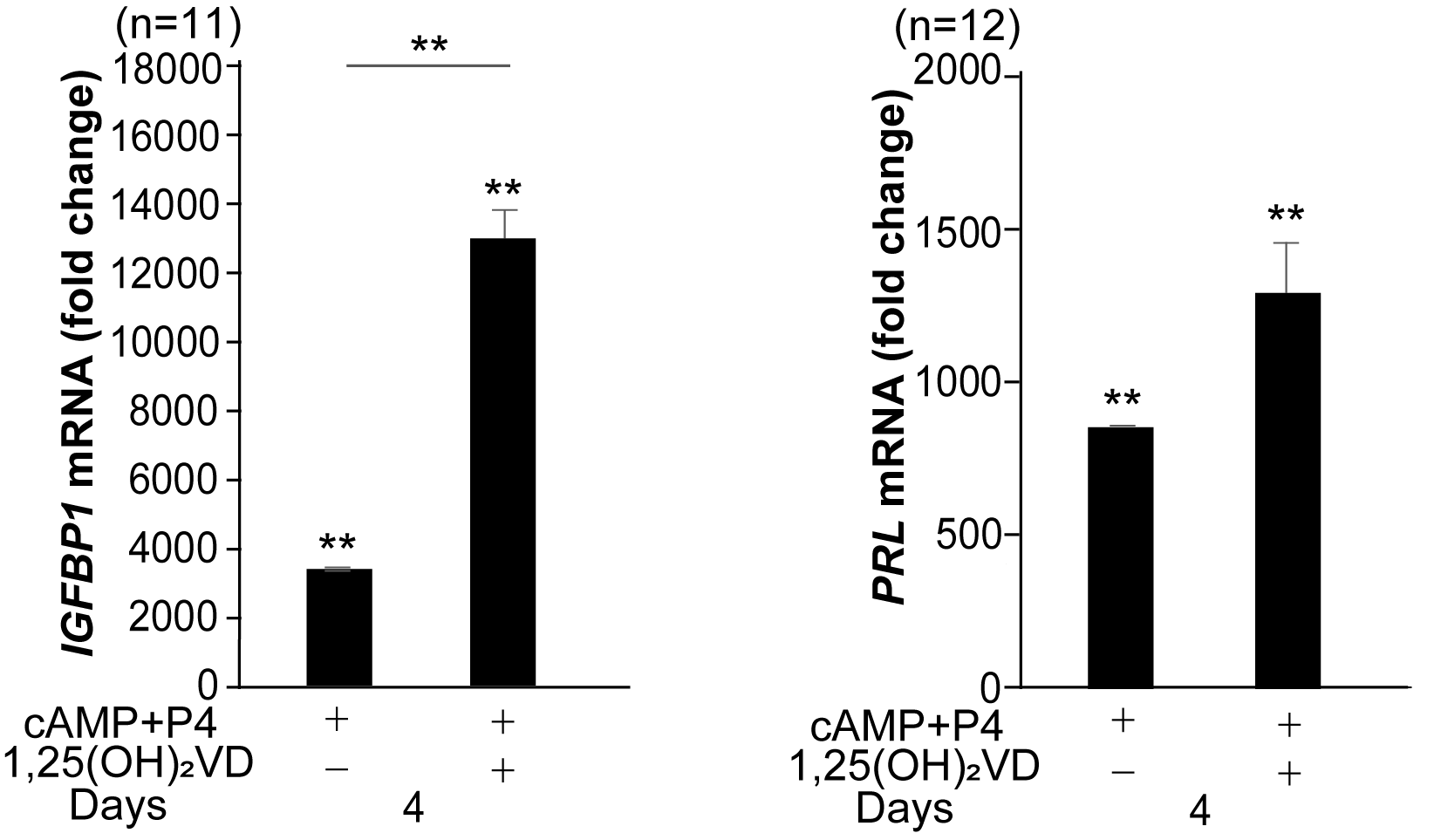

Supplement: Supplementary file 1 [file nutrients-10-00902-s001.zip › Supplemental fig. S1.tif]
